# Reevaluating sport tourism advancement in emerging destinations: a community-focused framework for sustainable value generation

**DOI:** 10.3389/fspor.2026.1795290

**Published:** 2026-03-06

**Authors:** Qodirov Azizjon Anvarovich, Urakova Dilfuza Bakhriddinovna

**Affiliations:** 1 Department of Accounting and Statistics, Bukhara State University, Bukhara, Uzbekistan; 2 Department of Tourism and Hotel Management, Bukhara State University, Bukhara, Uzbekistan

**Keywords:** community engagement, destination management, emerging markets, event legacy, post-pandemic tourism, resilience, silk road, social sustainability

## Introduction

The worldwide sport and event tourism business, currently valued at $1.41 trillion and anticipated to attain $2.12 trillion by 2032, offers exceptional prospects for nascent places pursuing economic diversification (1). Sport tourism accounts for 14% of foreign visitor arrivals, expanding at an annual pace of 8.2%, which is roughly double that of traditional tourism (2). The prevailing development paradigm, defined by infrastructure-driven mega-event strategies, has generated concerning counter-narratives: displacement in Rio de Janeiro, debt in Athens, and “white elephant” facilities in various host nations (3, 4).

This opinion post contends that growing destinations must fundamentally reevaluate sport tourism development, transitioning from infrastructure-focused strategies to community-oriented frameworks that regard communities as co-creators instead of passive benefactors. Utilizing evidence from Central Asian contexts and incorporating insights from contemporary research on mega-event legacies (5), sustainability transitions in tourism (6), and strategic communication (7), we assert that sustainable sport tourism necessitates the equilibrium of destination competitiveness with community welfare via systematic stakeholder engagement and benefit-sharing frameworks.

The imperative for paradigmatic reconsideration has been amplified by the COVID-19 pandemic, which exposed fundamental vulnerabilities in traditional sport tourism models predicated on maximizing visitor volumes and infrastructure investment. Pashaie and Perić (8) demonstrate that post-pandemic sport tourism development depends critically on “environmental forces and targeted support,” with successful strategies emphasizing safety, digitalization, and new employment opportunities rather than mere capacity expansion. Their paradigm model suggests that the industry's future lies in adaptive governance structures capable of responding to external shocks while maintaining community welfare—principles that align closely with the community-centered framework we propose. The pandemic thus serves not merely as a disruption but as a catalyst for reconceptualizing sport tourism development in emerging destinations.

Uzbekistan, Central Asia's most populous nation with 35 million inhabitants and a goal of attracting 15 million international tourists by 2030, exhibits the opportunities and problems confronting transitional economies. Uzbekistan has invested $2.3 billion in sports infrastructure since 2017 and has successfully hosted events such as the 2022 Central Asian Games and the 2023 World Wrestling Championships, showcasing a substantial commitment to the advancement of sport tourism (9). Nevertheless, accumulating evidence indicates that infrastructure alone cannot ensure lasting success without adequate focus on community integration and social license.

## Challenging the infrastructure-first paradigm

The dominant “event-led development paradigm” (10) posits that infrastructure investment stimulates extensive economic transformation, as demonstrated by Barcelona 1992, Manchester 2002, and Cape Town 2010. This paradigm, mostly created in established democracies with advanced institutional frameworks, may not be applicable to transitional economies characterized by distinct governance structures, development agendas, and socio-cultural contexts (11, 12).

Critical scholarship utilizing Social Exchange Theory indicates that community support is fundamentally contingent upon perceived benefit-cost ratios, exhibiting threshold effects beyond which acceptance diminishes swiftly (13, 14). Toyirova et al. (5) illustrate that mega-events can act as catalysts for sustainability enhancements in hospitality operations, albeit with significant disparities in implementation efficacy and legacy sustainability—results that highlight the critical role of institutional mechanisms in converting event investments into enduring advantages. Data from growing locations reveals crucial limits of around 15,000 daily visitors and 7-day event durations, beyond which community tolerance markedly declines, irrespective of economic advantages. This research indicates that the temporal and spatial concentration inherent in mega-events presents specific hazards for destinations without established coping strategies.

The thresholds cited above (15,000 daily visitors; 7-day event duration) should be understood as illustrative benchmarks derived from cross-case synthesis rather than universal limits. These figures emerge from integration of multiple sources: empirical studies of community attitudes in emerging tourism destinations, particularly research conducted in Southeast Asian and Eastern European contexts [see (14)]; policy guidelines from UNWTO's sustainable tourism indicators program; and comparative analysis of event impact assessments across transitional economies. Carrying capacity is inherently contextual, varying with existing tourism infrastructure, community experience with visitor flows, cultural attitudes toward outsiders, and local economic dependency on tourism. We recommend that destinations develop localized threshold assessments through participatory community research rather than adopting these figures as prescriptive standards. The benchmarks serve heuristic purposes—alerting planners to the existence of tolerance limits—rather than providing definitive numerical boundaries.

Furthermore, studies frequently reveal substantial discrepancies in perceptions of sport tourism impacts between industry stakeholders and local communities. Tourism operators and government officials highlight economic advantages and global exposure, whereas residents articulate significantly greater apprehensions regarding congestion, price inflation, cultural commodification, and the hazards of gentrification. These differing perspectives highlight the necessity of inclusive planning procedures that authentically include community input instead of regarding citizens as impediments to development.

Before presenting our proposed framework, it is essential to articulate its relationship to established bodies of scholarship and clarify its conceptual contribution. Three theoretical traditions inform our approach: community-based tourism (CBT) models, legacy planning frameworks, and social license to operate (SLO) concepts. We position the Sustainable Sport Tourism Assessment Framework (SSTAF) as an integrative reconfiguration that extends, synthesizes, and adapts these approaches specifically for sport tourism development in emerging destinations.

Community-based tourism models (15, 16) emphasize local participation, benefit distribution, and empowerment as prerequisites for sustainable tourism development. While these principles are foundational to our approach, traditional CBT frameworks were developed primarily for nature-based and cultural tourism contexts characterized by continuous, relatively stable visitor flows. SSTAF extends CBT principles by incorporating sport-specific elements absent from conventional models: event temporality (the concentrated, episodic nature of sport tourism), venue utilization cycles (the challenge of maintaining community benefit between events), and athlete-community interactions (the unique social dynamics generated by elite sport). These extensions address the distinctive challenges posed by sport tourism's cyclical intensity patterns.

Legacy planning frameworks (17, 18) provide sophisticated tools for evaluating mega-event outcomes across multiple dimensions. However, these frameworks have been criticized for their predominantly *post-hoc* evaluative orientation and their development within contexts of established institutional capacity. SSTAF integrates legacy planning with real-time threshold monitoring, transforming legacy considerations from retrospective assessment to proactive impact management. This temporal reorientation is particularly critical for emerging destinations that lack the institutional buffers to absorb planning failures.

Social license to operate concepts (19), originally developed in extractive industries, have been increasingly applied to tourism contexts to understand community acceptance dynamics. Conventional SLO applications treat social license as a risk-management concern—something to be monitored and maintained to prevent opposition. SSTAF reconfigures SLO from a reactive risk-management tool to a foundational design principle that shapes development trajectories from inception. Rather than asking “how do we maintain community acceptance for our plans,” the framework asks “how do community aspirations and thresholds shape what plans are appropriate.”

The integrative reconfiguration embodied in SSTAF thus offers more than contextual application of established principles. It provides a conceptual architecture that synthesizes complementary insights while addressing the specific challenges of sport tourism in emerging destination contexts—contexts characterized by institutional fragility, limited tourism experience, and heightened vulnerability to development-induced disruption.

## A community-centered framework for emerging destinations

**Figure 1:**
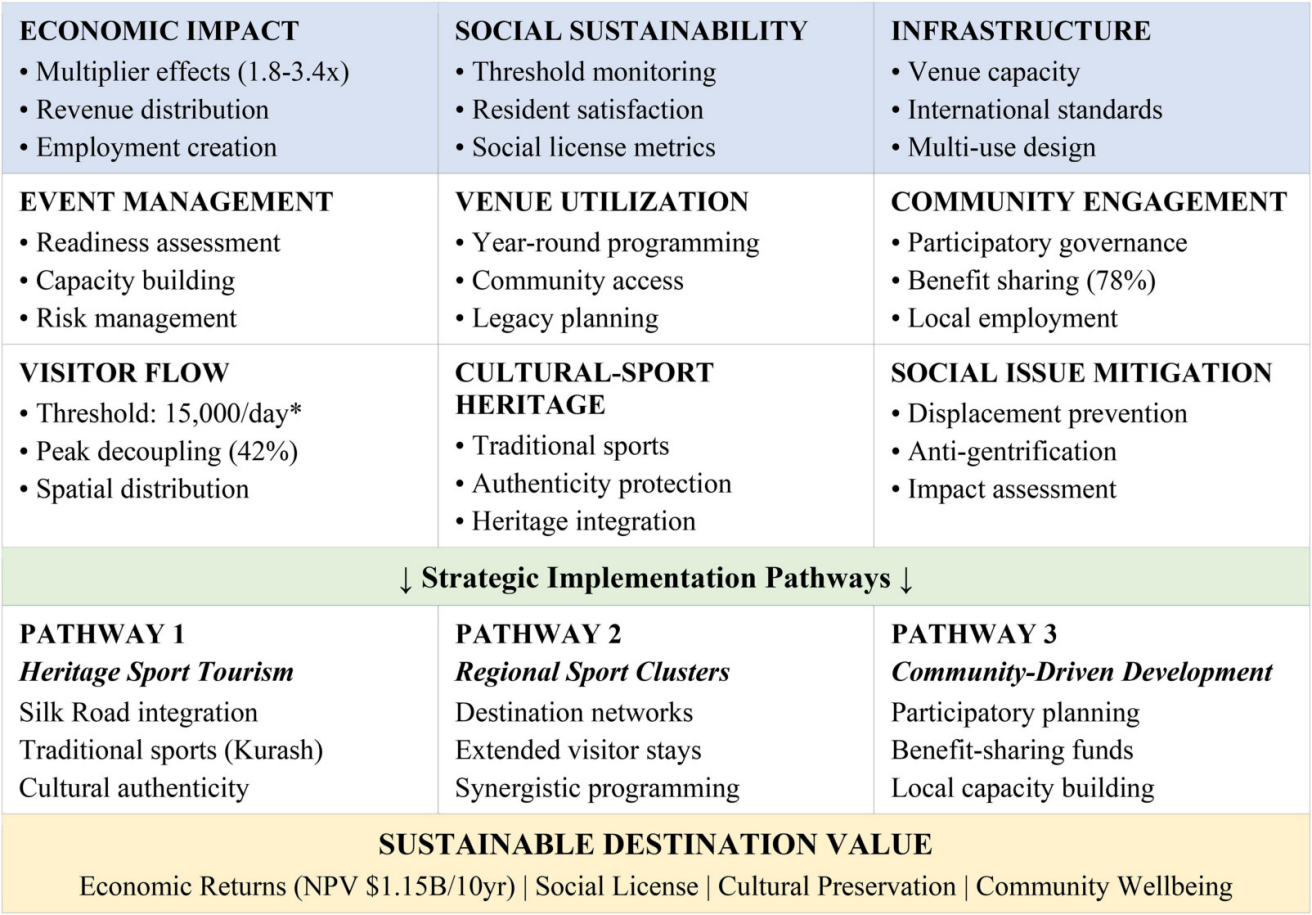
Sustainable sport tourism assessment framework (SSTAF) for emerging destinations. Developed by the authors based on sustainable tourism theory, community engagement frameworks, and sport event legacy research.

Following stakeholder consultation and comparative research, we propose the Sustainable Sport Tourism Assessment Framework (SSTAF), which consists of nine structural elements crucial for equitable growth in growing destinations. Figure 1 depicts the architectural structure and the interrelations among components.

The SSTAF emphasizes community engagement as its primary organizing principle, aligning with accumulating evidence that benefit-sharing systems and participatory governance yield more stakeholder acceptance (78% approval rates) compared to infrastructure-centric strategies. This discovery questions traditional beliefs that venue construction is the main factor in sport tourism success, indicating that social license serves as a limiting factor on sustainable development.

### Temporal dynamics and framework operation

The nine framework elements operate dynamically across three temporal phases, with community engagement serving as both the foundation upon which the framework rests and the thread that weaves through all temporal phases. Understanding these temporal dynamics is essential for practical implementation.

#### Pre-event phase

During the planning and development stage, community engagement establishes the foundational conditions through participatory governance structures and negotiated benefit-sharing agreements. Infrastructure planning occurs within parameters set by community consultation, emphasizing multi-use design and community access provisions. Social issue mitigation activities—displacement prevention protocols, anti-gentrification measures, and baseline impact assessments—are implemented proactively. Cultural-sport heritage elements are identified and protection mechanisms established. This phase determines the carrying capacity thresholds that will guide event-time management.

#### Event-time phase

During active events, visitor flow management operationalizes the thresholds established through pre-event community consultation, with real-time monitoring triggering adaptive responses when daily visitor numbers or spatial concentrations approach agreed limits. Event management protocols activate capacity implementation plans and risk management procedures. Cultural-sport heritage protection ensures authenticity maintenance during periods of intensive visitor contact. Community engagement continues through feedback mechanisms and rapid-response systems for addressing emergent concerns.

#### Post-event phase

Following events, venue utilization programming ensures year-round community access and prevents facilities from becoming isolated assets. Economic impact assessment tracks multiplier distribution and employment sustainability against pre-event commitments. Social sustainability monitoring measures resident satisfaction and determines whether social license has been maintained, enhanced, or eroded. Legacy planning incorporates lessons learned into future event bidding and development decisions. Community engagement in this phase focuses on accountability—ensuring that promised benefits materialized and addressing any shortfalls.

#### Community engagement as cross-cutting principle

It is essential to clarify that community engagement operates simultaneously as a discrete framework element and as a cross-cutting principle that conditions all other elements. As a discrete element, it encompasses specific activities: participatory governance structures, benefit-sharing mechanisms, and local employment programs. As a cross-cutting principle, it shapes how all other elements are conceptualized and implemented—infrastructure decisions are made with community input, threshold monitoring reflects community-defined tolerance levels, and legacy planning responds to community-articulated priorities. This dual character means that community engagement is both a sequential entry point (it must be established before development proceeds) and an omnipresent consideration (it informs every subsequent decision).

The framework delineates three implementation pathways specifically tailored for emerging locations. Heritage Sport Tourism utilizes cultural assets—traditional sports like Kurash wrestling and Silk Road linkages—to distinguish its offers from established rivals. Regional Sport Clusters link complementing sites to prolong visitor stays and disseminate advantages across regions. Community-Driven Development guarantees that local residents engage substantively in planning, implementation, and benefit distribution. The paths are not mutually exclusive; ideal techniques presumably integrate features from all three.

## Implications for policy and practice

The research advocates for multiple strategic adjustments for policymakers in emerging destinations. Mandatory social impact assessments should be conducted prior to every event with over 5,000 attendees, along by threshold monitoring systems to track daily visitor numbers and community opinion. Secondly, Community Benefit Funds funded by event levies can guarantee that concrete returns are distributed to local residents instead of being concentrated among industry interests. Third, capacity building must precede mega-event bidding—cultivating human capital, governance frameworks, and community involvement strategies prior to seeking prestigious international contests.

The framework highlights the necessity for destination managers to implement year-round venue programming to enhance utilization rates, which now average a mere 34% yearly, and to guarantee that facilities address community needs beyond occasional events. Strategic communication that frames sport tourism as a means for community development, rather than solely for economic gain, can improve social license and diminish resistance (7, 20). Toyirova et al. (5) assert that the sustainability legacies of mega-events are “complex, context-dependent, and not universally positive,” emphasizing the necessity for destination-specific measures instead of generic solutions. The incorporation of sustainability concepts from relevant industries, including insights from energy efficiency transitions as recorded by Toyirova (6), can guide long-term planning strategies that reconcile short-term gains with intergenerational equity.

Our framework responds directly to the post-pandemic imperative identified by Pashaie and Perić (8) for sport tourism to operate within new paradigmatic assumptions that prioritize resilience and sustainability over growth maximization. Their emphasis on “digitalization” aligns with our framework's threshold monitoring systems, which can leverage digital technologies for real-time visitor tracking and community sentiment analysis. Their focus on “new employment opportunities” connects to our community benefit provisions that prioritize local employment creation and skill development over imported labor. The post-COVID context thus reinforces rather than challenges our community-centered approach: destinations that embed resilience through strong community relationships will be better positioned to weather future disruptions than those dependent on infrastructure assets alone.

The framework provides researchers with a diagnostic tool suitable for various developing market environments, featuring modular components that can be tailored to specific institutional and cultural situations. Future research should explore the long-term effects of community-centered strategies, comparative results across various pathway combinations, and the mechanisms by which social license converts into sustained competitive advantage.

## Conclusion

The global sport tourism sector offers transformative prospects for emerging places; nevertheless, achieving sustained value necessitates a comprehensive reevaluation of prevailing development assumptions. The infrastructure-first strategy that defined twentieth-century sport tourist development—frequently resulting in impressive facilities, displaced communities, and mounting debt—serves as a cautionary model that transitional economies should deliberately eschew.

The proposed community-centered framework regards citizens not as passive beneficiaries or impediments to development, but as vital co-creators whose involvement influences long-term sustainability. Evidence from emerging destinations indicates that community benefit programs, participatory governance, and heritage integration yield greater stakeholder acceptance than infrastructure investments alone, thereby challenging the prevailing notion that venue construction is the primary determinant of success.

As regions in Central Asia and beyond seek to develop sport tourism, they confront a critical decision: to emulate unsustainable models that emphasize spectacle over substance, or to forge innovative strategies that harmonize economic advancement with social accountability and cultural conservation. The research indicates that the latter approach, although necessitating increased patience and institutional capability, provides enhanced opportunities for sustainable value generation. The definitive criterion for assessing the success of sport tourism should transcend mere visitor numbers and medal tallies, incorporating community prosperity, cultural vibrancy, and intergenerational health.

## References

[1] UNWTO. Sport Tourism and Sustainable Development Goals. Madrid: World Tourism Organization (2023).

[2] Getz D Page SJ. Progress and prospects for event tourism research. Tour Manag. (2016) 52:593–631. 10.1016/j.tourman.2015.03.007

[3] Müller M. The mega-event syndrome: why so much goes wrong in mega-event planning. J Am Plan Assoc. (2015) 81(1):6–17. 10.1080/01944363.2015.1038292

[4] Gaffney C. Gentrifications in pre-Olympic Rio de Janeiro. Urban Geogr. (2018) 37(8):1132–53. 10.1080/02723638.2015.1096115

[5] Toyirova SA Canós-Darós L Osorio-Acosta E. Green and resilient hotel operations through mega-event legacies. Front Sports Act Living. (2025) 7:1604131. 10.3389/fspor.2025.1604131 40895404 PMC12394522

[6] Toyirova H. Long-term decision-making in energy efficiency management: evidence from Uzbekistan. Front. Energy Effic. (2025) 3:1606823. 10.3389/fenef.2025.1606823

[7] Radjabov O Davronov IO Boltayeva M Ashurova M Navruz-zoda L. Prospects of using strategic communication in sustainable tourism promotion. Front Sports Act Living. (2025a) 7:1623121. 10.3389/fspor.2025.1623121 41306186 PMC12644039

[8] Pashaie S Perić M. The future of sports tourism in the light of the COVID-19 pandemic – developing a new paradigm model. J Tour Futures. (2025) 11(3):390–405. 10.1108/JTF-09-2022-0236

[9] Government of Uzbekistan. Development Strategy of New Uzbekistan 2022-2026. Tashkent: State Publishing (2022).

[10] Roche M. Mega-events and Modernity: Olympics and Expos in the Growth of Global Culture. London: Routledge (2000).

[11] Nauright J Pope S. The twenty-first-century SportsWorld: global markets and global impacts. Sport Soc. (2017) 20(3):289–98. 10.1080/17430437.2017.1234014

[12] Koch N. The geopolitics of sport beyond soft power. Sport Soc. (2018) 21(12):2010–31. 10.1080/17430437.2018.1487403

[13] Ap J. Residents’ perceptions on tourism impacts. Ann Tour Res. (1992) 19(4):665–90. 10.1016/0160-7383(92)90060-3

[14] Kaplanidou K Karadakis K Gibson H Thapa B Walker M Geldenhuys S Quality of life, event impacts, and mega-event support among South African residents. J Travel Res. (2016) 52(5):631–45. 10.1177/0047287513478501

[15] Murphy PE. Tourism: A Community Approach. London: Methuen (1985).

[16] Scheyvens R. Ecotourism and the empowerment of local communities. Tour Manag. (1999) 20(2):245–9. 10.1016/S0261-5177(98)00069-7

[17] Preuss H. The conceptualisation and measurement of mega sport event legacies. J Sport Tour. (2007) 12(3-4):207–28. 10.1080/14775080701736957

[18] Chalip L. Towards social leverage of sport events. J Sport Tour. (2006) 11(2):109–27. 10.1080/14775080601155126

[19] Thomson I Boutilier RG. Social license to operate. In: Darling P, editor. SME Mining Engineering Handbook. Englewood, CO: Society for Mining, Metallurgy and Exploration (2011). p. 1779–96.

[20] Radjabov O Gubíniová K Vilčeková L Remiaš K. Analysis of the most common mass communication tools of marketing communication respecting the criteria of sustainability within tourism industry. In Developments in Information and Knowledge Management Systems for Business Applications. Cham: Springer (2025).

